# Comparative Analysis of V-Akt Murine Thymoma Viral Oncogene Homolog 3* (AKT3)* Gene between Cow and Buffalo Reveals Substantial Differences for Mastitis

**DOI:** 10.1155/2018/1463732

**Published:** 2018-05-15

**Authors:** Farman Ullah, Dinesh Bhattarai, Zhangrui Cheng, Xianwei Liang, Tingxian Deng, Zia Ur Rehman, Hira Sajjad Talpur, Tesfaye Worku, Rahim Dad Brohi, Muhammad Safdar, Muhammad Jamil Ahmad, Mohammad Salim, Momen Khan, Hafiz Ishfaq Ahmad, Shujun Zhang

**Affiliations:** ^1^Key Laboratory of Agricultural Animal Genetics, Breeding and Reproduction, Education Ministry of China, College of Animal Science and Technology, Huazhong Agricultural University, Wuhan 430070, China; ^2^Faculty of Veterinary and Animal Sciences, Department of Animal Breeding and Genetics, Lasbela University of Agriculture, Water and Marine Sciences, Uthal, Balochistan, Pakistan; ^3^Department of Pathobiology and Population Sciences, Royal Veterinary College, Hawkshead Lane, Hatfield, Hertfordshire AL9 7TA, UK; ^4^The Opening Project of Guangxi Key Laboratory of Buffalo Genetics, Reproduction and Breeding, Nanning, Guangxi 530000, China; ^5^Department of Animal Health, Faculty of Animal Husbandry and Veterinary Sciences, The University of Agriculture, Peshawar, Pakistan; ^6^Department of Forestry and Wildlife Management, The University of Haripur, Khyber Pakhtunkhwa, Pakistan; ^7^Livestock and Dairy Development, Khyber Pakhtunkhwa, Pakistan

## Abstract

*AKT3* gene is a constituent of the serine/threonine protein kinase family and plays a crucial role in synthesis of milk fats and cholesterol by regulating activity of the sterol regulatory element binding protein (SREBP).* AKT3* is highly conserved in mammals and its expression levels during the lactation periods of cattle are markedly increased.* AKT3* is highly expressed in the intestine followed by mammary gland and it is also expressed in immune cells. It is involved in the TLR pathways as effectively as proinflammatory cytokines. The aims of this study were to investigate the sequences differences between buffalo and cow. Our results showed that there were substantial differences between buffalo and cow in some exons and noteworthy differences of the gene size in different regions. We also identified the important consensus sequence motifs, variation in 2000 upstream of ATG, substantial difference in the “3′UTR” region, and miRNA association in the buffalo sequences compared with the cow. In addition, genetic analyses, such as gene structure, phylogenetic tree, position of different motifs, and functional domains, were performed to establish their correlation with other species. This may indicate that a buffalo breed has potential resistance to disease, environment changes, and airborne microorganisms and some good production and reproductive traits.

## 1. Introduction

Buffalo are more defiant to disease and antagonistic surroundings compared with cows [1–7]. In the present studies, therefore, we focused on investigating the differences in genomic sequences between buffalo and cow. Livestock are the main source of animal proteins such as meat, fish, poultry, eggs, and dairy products. They are also considered as the source of draft power in human daily agricultural activities in some areas. In human civilization history, cattle play a vital role while the cattle phylogeny is still debatable. Cattle were the first livestock animal whose full genome has been mapped [8]. The human whole genome sequence and its importance in the genetic complications have been established. This has contributed to the understanding of the phenotype diversity and disease and provided an approach to sequencing the whole genome of mammals and showing their correlation with human (cattle, buffalo, dogs, pigs, and cats) [9].

AKT family plays key roles in mammary gland development, lactation, and degradation, and their isoforms are potential candidate genes associated with milk production even though they have some different function [10]. With the advance in new biotechnology and science, it is important to review the genetic variability among and within different species. This measurement could be very supportive for preserving genetic resources.

Buffalo is an important domestic animal in subtropical and tropical areas. However, the mechanism of genes related to mammary gland growth, lactation, and deprivation of lipid metabolism is not fully understood. AKT family is involved in a multiplicity of genetic processes such as counting cell propagation, demarcation, angiogenesis, apoptosis, tumor genesis, metabolism, cell survival, growth, glycogen synthesis, and glucose uptake [11, 12].* AKT3* is well expressed in immune cells and this suggests its involvement in immune process [13]. AKT genes play important roles in mammary gland maturity, lactation, dilapidation, and lipid amalgamation and this indicates its importance in milk production [14]. Asian buffalo has promising characteristics for production as a livestock species with socioeconomic importance. In developing countries the buffalo is mostly used for providing milk and meat for local communities through integrating supply systems.

The buffalo milk has some intrinsic characteristics such as high fat contents which are favorable for cheese production. For example, Mozzarella cheese in Italy is a famous dairy product from buffalo milk. The genomic data resources of buffalo, a “subsequent kith and kin” species of cattle, are not fully established. In regard to evolution, the buffalo and cattle species have close rapport. In comparison to buffalo, the cattle genome is far better characterized. Derived markers of cattle were used initially for buffalo genome maps and potential rearrangements were identified between those species [15]. Nonetheless, the application of cattle genomic tools to buffalo is not frank and, as shown in the outcome, regardless of genome sequences resemblance, the genetic polymorphisms are diverse [16].

The AKT serine/threonine protein kinase, also branded as (PKB) protein kinase B, was reported as the protooncogene v-akt homolog in 1991 [14, 17]. There are three subtypes of AKT in mammals:* AKT1*,* AKT2*, and* AKT3*, which are preset by 3 different genes independently, AKTs are the downstream effectors of the PI3K signaling alleyway [18, 19]. To regulate the glucose metabolism balance the PI3K-AKT signal pathway performs a decisive function [20]. Previous studies have reported that, in the fibroblast cell line in mouse, the ATP levels are substantially influenced by the AKT family [21, 22]. Appropriate maturity in bovine mammary tissue is necessary for high milk production. It is an important factor affecting milk production traits.

Therefore, for proper mammary gland development, it is essential to provide appropriate energy supplies which are essential for lactation, degradation, and proper mammary gland development. It is yet not clear how the AKT regulates the above processes in the mammary gland. The AKT expression in mice during late pregnancy was substantially upregulated [23]. Despite functions specific differences in AKT isoforms, all 3 subtypes are imminent entrant genes linked with milk production. A recent study showed that, during lactation periods in cattle, the expression levels of* AKT1* and* AKT3* were markedly increased [10]. In addition, the synthesis of breast milk fat and cholesterol are affected by the changes in AKT. AKT family contributes to numerous disorders such as inflammation and ischemia [24]. Experimental autoimmune encephalomyelitis, which plays a role in central nervous system and immune system susceptibility, is regulated by* AKT3*. The previous studies show that the isotope* AKT3* of the AKT family is involved in various functions. We hypothesized that, compared with cows, buffalo have more potential characteristics toward resistance to mastitis disease and adverse environmental conditions, which may account for these species unambiguous differences.

Our bioinformatics analysis unraveled a few but actually decisive, preset, and noteworthy differences of* AKT3* gene between buffalo and cattle. The results of this study will provide important information to strength and elicit genome structure, metabolism, and physiology differences between species and their evolution.

## 2. Materials and Methods

### 2.1. In Silico and Bioinformatics Analysis

#### 2.1.1. Data Structure

The* AKT3 *gene sequences of buffalo (NW_005783781.1) and cattle (AC_000173.1) were selected from the database of National Center for Biotechnology Information (NCBI https://www.ncbi.nlm.nih.gov/gene/?term=akt3) [25]. Bioinformatics approach was used to target the 5′-upstream region, 5′ UTR region, coding regions, and “3′UTR” region for the differences in the* AKT3* gene between buffalo and cattle. The 5′-upstream region and “5′UTR” region are important for the promoter sequences, transcription starting sites (TSS), and CPG island prediction and differences in buffalo and cow. The coding region is important for the single nucleotides polymorphism sites. The “3′UTR” region is concerned with the prediction and differences of miRNA in buffalo and cattle. The bioinformatics tools including fruit fly, cbs, ebi, mirbase, smart, and pfam were used for different regions of* AKT3* differences between buffalo and cow.

### 2.2. Promoter Prediction, Transcription Starting Sites (TSS), and CPG Island Prediction

The promoter sequences prediction and differences identification in buffalo and cow were carried out using the bioinformatics tools in http://www.fruitfly.org/seq_tools/promoter.html. The differences in the consensus sequences motifs in the promoter region might cause changes in the expression of gene in different species and their specific functions. For the transcription starting sites prediction and differences in buffalo and cow, the tool in http://www.cbs.dtu.dk/services/Promoter/ was used. These consensus sequences motifs have key role in the gene transcription and expression. The CPG islands in buffalo and cow were predicted using MethPrimer (http://www.urogene.org/methprimer/) and the tools provided in https://www.ebi.ac.uk/Tools/emboss/cpgplot/ [26].

### 2.3. Sequences Similarity, miRNA Prediction, and Protein-Protein Interaction

For the sequences similarity and mRNA analysis, we performed the highly similar sequence (megaBLAST) analysis. NCBI blast tools were used (https://blast.ncbi.nlm.nih.gov/Blast.cgi). The tool of mirbase (http://www.mirbase.org/search.shtml) was used for the prediction of miRNA in “3′UTR” region in buffalo and cow. We used an NCBI blasting tool (https://blast.ncbi.nlm.nih.gov/Blast.cgi) to compare the differences between buffalo and cow in their “3′UTR” region. We also used research tool for protein-protein interaction for the buffalo and cow provided in https://www.string-db.org/. It was also used to determine the proteins and protein interactions in both buffalo and cows.

### 2.4. Protein Structure and Domains Prediction

The in silico analysis was carried out for the protein structure and domain prediction in both buffalo and cow. The different bioinformatics research tools were used, including http://smart.embl-heidelberg.de/smart/change_mode.pl, http://smart.embl-heidelberg.de/help/smart_about.shtml [27], http://pfam.xfam.org/search/sequence [28], https://www.ebi.ac.uk/services, https://prosite.expasy.org/, and https://www.ncbi.nlm.nih.gov/Structure/cdd/wrpsb.cgi.

### 2.5. Alignment, Phylogenetic Tree, Motifs and Gene Structure, Proximal Control, and Core Promoters

The alignments and phylogenetic trees were performed in twenty different species to establish their association and evolutionary relationship with these animals for the same gene. The evolutionary tree is designed by MEGA6 or fig tree in Gene Doc software [29]. The gene structure display server program (GSDS) was used for gene structure analysis [30]. To display the motifs in these species and analyze the proximal control elements and core promoters, we used the MEME 4.10.1 program [31]. GC box or GSG box is a nucleotides distinct pattern regulatory transcriptional cis-acting element in the promoter region. The proximal control elements restrain GC box (GGGCG) and CAAT box (GCCCAATCT). The core promoters included TSS and TAATA box (TATAAA).

## 3. Results and Discussion

The* AKT3* size is 287 kb and 265 kb in buffalo (locus NW_005783781, 286082 bp) and cow (locus NC_007314, 267865 bp), respectively, and they are located on chromosome 16. AKT in humans and mice were adaptable bustle of the sterol regulatory element binding protein (SREBP), which influences the production of breast milk fat and cholesterol [32–35]. In regard to mammary gland of mice, regulation of SREBP by AKT is through two possible pathways, both of which escort to boost in nuclear SREBP.

AKT family is involved in a variety of biological processes. AKT plays a role in relocation of juvenile SREBPS through promoting the coat protein toward the golgi from the endoplasmic reticulum and/or inhibiting the glycogen synthesis kinase-3*β* phosphorylation [36]. Integrity of CNS cell and T cell function regulation require the presence of* AKT3*.* AKT3* is expressed in immune cells [13]. For controlling cell number and size, the* AKT3* was required [37]. In the PI3K pathway AKT isotopes are the crucial signaling molecules which regulate cell growth, proliferation, survival, and metabolism [13]. For tackling diseases, their immunity related functions are important [38, 39]. AKT is also involved in the regulation of inflammatory cytokines with proinflammatory property as shown in the TLR signaling pathway. The functional important genes related to TLR pathway contain markers in breeding selection [40].

### 3.1. Cow and Buffalo Sequences Similarity

**Figure 1:**
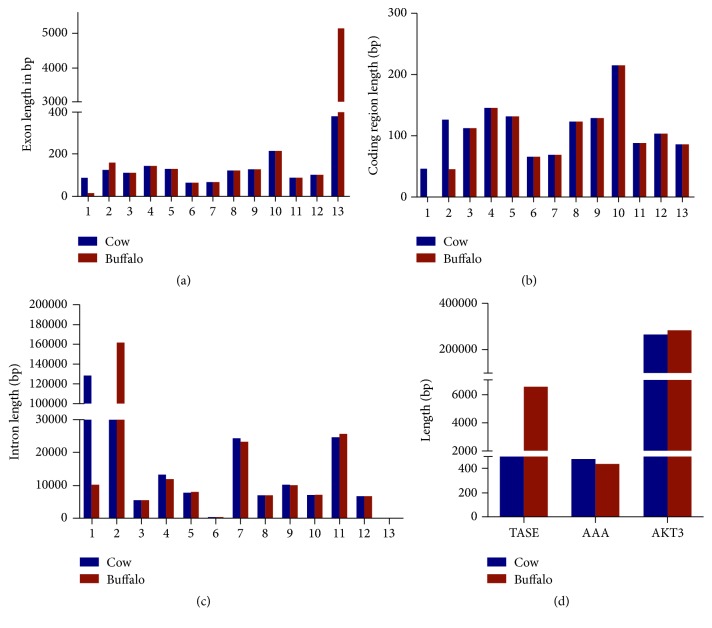
Length of* AKT3* gene, exons, and introns in both cow and buffalo. (a) Cow and buffalo 13 exons length (bp). (b) Comparing cow and buffalo coding length (bp) of 13 regions. (c) Intron length (bp) of cow and buffalo. (d) Total length of annotated spliced exon of* AKT3*, annotated amino acids, and* AKT3* gene for cow and buffalo.

We have analyzed the sequencing data of cattle to identify the SNPs through the databases from NCBI and Ensembl. Data in Table 1 were synchronized with the SNPs list for measurement and analysis. The buffalo SNPs data are not available in any database for harmonization with the relevant SNPs records. In Figure 1, the typescript of* AKT3* for cow and buffalo in interval (exons 5′ to 3′) is available in databases. In Figure 1(a), there are 13 exons in cow and buffalo which show substantial differences in the cow and buffalo in exon size in exons numbers 1, 2, and 13.

The coding lengths between cow and buffalo are different in the 1 and 2 coding regions. There is no coding sequence in region 1 in buffalo while the 2 coding regions in cow are longer than those in buffalo, indicating substantial differences. The intron size shows substantial differences on region 1 and 2. In the exon number 13, no coding regions were detected in both species. In region 1, the intron size in cow is four times that of buffalo, while on region 2, in the buffalo, the intron size is two times larger than that in cow. Total size of annotated spliced exon in the buffalo is threefold larger than that in the cow while there are more annotated amino acids in the cow, indicating substantial differences. The total gene size followed similar patterns as above. In the cow, it is sized at 265 kb while in the buffalo its size is 287 kb, which shows that buffalo have a larger size of* AKT3* gene. The sequence similarity in important regions is strong indication of common ancestor. These sequences are related to evolution of divergence. We, therefore, used bioinformatics tools to find out the important regions of similarity and arranged different sequences of RNA, DNA, and protein. This is important for establishing the structural evolutionary relationships and functions between these sequences. To focus on the differences of sequences between cow and buffalo, we analyzed the cross similarity of exons in cow and buffalo. We have found some important differences in the exon regions. RNA expression with spatial distribution constitutes molecular characterization of a gene. Transcription of RNA into cDNA genetic information is the first step in gene expression. For confirmation of the result, we also analyzed the mRNA in both cow and buffalo and found the same differences.

A total of 13 exons were found in the cow and buffalo for* AKT3* gene. We performed the high similarity sequence (megaBLAST) analysis of the exon one by one between cow and buffalo using NCBI blast tool (https://blast.ncbi.nlm.nih.gov/Blast.cgi). In total, we found the substantial differences in 6 out of 13 exons between cow and buffalo. The important differences in exons 4, 6, 7, 9, 12, and 13 have been found between cow and buffalo. Each of the first 5 exons has one amino acid difference while exon 13 has four amino acid differences in its sequence between cow and buffalo. To confirm these findings, we carried out the high similarity sequence (megaBLAST) analysis for the mRNA between cow and buffalo using an NCBI blast tool, (https://blast.ncbi.nlm.nih.gov/Blast.cgi?PROGRAM=blastx&PAGE_TYPE=BlastSearch&LINK_LOC=blasthome). This derived the same result we have obtained in the exons (Table 2). Our study highlighted the substantial differences of* AKT3* gene between the cow and buffalo.

In addition to determination of the substantial differences in cow and buffalo for* AKT3 *gene, our results suggest that there is a difference in position for the genome between buffalo and cow, which may be important for coexpression of the gene. These findings provided information for enhancing tolerance toward disease, stress, and so forth although they may differ between genotypes.

### 3.2. Prediction of Promoters, Proximal Control, Core Promoters, and Other Consensus Sequence Motifs

Gene network analyses assist in the classification of genes that have pleiotropic effects and/or regulatory roles [41]. The environments and their change in adopting new function of genes have important correlation as the genes were commonly diversified during evolution period of multigene families, which facilitates the evolutionary cooption of genes [42]. For the measurement accuracy, we took 2000 bp of the upstream from the starting ATG of the* AKT3* gene of cow and buffalo. The data of important sequence motifs of cow and buffalo are given in Table 3. It was interesting to note that the ATG in cow starts at the 1st exon while in the buffalo it starts from the second exon. The TATA was found in five different regions in the cow sequence, while in the buffalo sequence, it was as double as that of the cow. Analysis of type of DNA promoter sequences helps identify other molecules for starting transcription and the positions where the genetic sequence can be read and decoded. The TATAA was found in the cow in six different regions while in the buffalo it was found in two different regions only. The TATAAA was found in two different positions in the cow sequence while it was absent in the buffalo sequence. The TATAA changed in the cow sequence found in 15 different regions with change of TAT>TNT as G, T, and C were counted as 7, 6, and 2, respectively. In the buffalo its G, T, and C counts were 6, 6 and 2, respectively, in 14 regions. DNA sequence sandwiching is known as floxing between two loxP sites. The cyclic adenosine monophosphate responsive elements (CRE) occur in cow at two different regions with C>G and A. These are found in three different regions in buffalo sequence with C>G and A, with 2 and 1 times occurring, respectively. Enhancer box (CANNTTG) is also very important in eukaryotes as it acts as binding molecule with AST2. Enhancer box (E-box) is a short region of DNA and play keys roles in the regulation of gene expression in tissues such as muscles, neurons, and others acting as protein binding sites. In both cow and buffalo, there are two E-boxes in the same position while the other is located at different position. We showed the AG, GT, and GA, in occurrence of the NN replacement. The enhancer core GTGG is also available in the cow and buffalo sequences at different positions and repeats 7 times in sequences of both species. A DNA sequence of cis-regulatory is known as enhancer core (EC) and plays key role in gene expression with cluster of transcription. EC is important in the development of thymocyte and macrophage as observed in the transgenic studies of animal. It is expressed in a variety of tissues [43]. Nucleotides sequence has distinct patterns and is involved in regulatory process, which is known as CAAT box. CAAT box was found in both species at different positions. NF-KB (nuclear factor-kappa B) is involved in many normal cellular and organismal processes, such as developmental process, immune and inflammatory response, and apoptosis. NF-KB sequence (GGGRNNYYCC) is found in the species at different positions. It appeared five times in cow and two times in buffalo sequences with substantial variation. Interferon regulatory factor having N-terminal is located at a conserved region and plays a key role in activation or repressor of transcription. The sequence of IRF (interferon regulatory factor 3, GAAANNGAAAG) is also found with substantial variation in position and sequences between cow and buffalo.

Transcription factors SP1/specificity protein plays a key role in transcription activation or repressing. The sequence motifs of sp-1 (special protein binding site; sp-1 GGGCGG) occurred five times in cow and six times in buffalo sequence with important variation in position. Transcription starting site (TSS) occurred two times in the sequences of cow and buffalo at different positions. Transcription starting sites are near to the promoters which help with initiation of transcription for a particular gene. No CPG island is found in the sequences of cow and buffalo. CPG island is the DNA short stretch which is found in the upstream regions, where the sequence CG frequency is higher than others and greater than 50%, and ranges are counted when Obs/Exp value is greater than 0.6. The promoters are predicted in this region at two positions with one in the same position and the other in different region and substantial variation with G>T. These important consensus sequences motifs and variation observed for the promoter of cow and buffalo are shown in Table 3.

### 3.3. The Importance of Upstream Sequences and Their Variation between Cow and Buffalo

The important genes under study might be expressed, with substantial high variation in the starting ATG to upstream sequences in cow and buffalo. We observed and analyzed them with NCBI database (https://blast.ncbi.nlm.nih.gov/Blast.cgi?PAGE=MegaBlast&PROGRAM=blastn&BLAST_PROGRAMS=megaBlast&PAGE_TYPE=BlastSearch&BLAST_SPEC=blast2seq&DATABASE=n/a&QUERY=&SUBJECTS) and found some noteworthy variation in both species. They are presented in Table 4. Sequencing blast results of the cow and buffalo show 98% similarity but with variations at 40 positions of alleles. These may be associated with the transcription initiation and gene expression in different species with sequences variation and their function regulation.

### 3.4. miRNA Prediction

**Figure 2:**
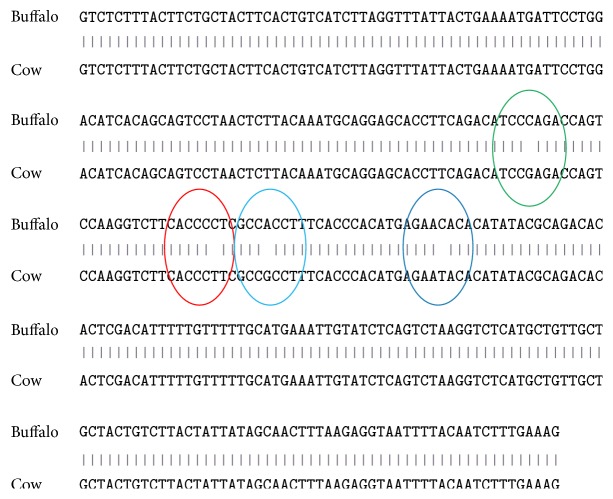
Comparing “3′UTR” of cow and buffalo and their variation observed in the sequences.

The “3′UTR” regions of the cow and buffalo were analyzed and some variations were detected. miRNA is small class of endogenous non-protein-coding RNAs that play important role in degradation/translation repression, RNA silencing, pathway synthesis, and regulatory process. The “3′UTR” regions in the cow and buffalo are also analyzed for miRNA prediction; the results are illustrated in Table 5 and variation is illustrated in Figure 2. These predictions will explore the variation and differentiation between cow and buffalo for gene function and expression. The “3′UTR” region in cow is 295 bp while it is much bigger in buffalo, 5072 bp. Translation termination codon follows immediately by section of messenger RNA (mRNA) and there are three prime untranslated regions (“3′UTR”) in molecular genetics.

### 3.5. Protein-Protein Interaction, Structure, and Domain

**Figure 3:**
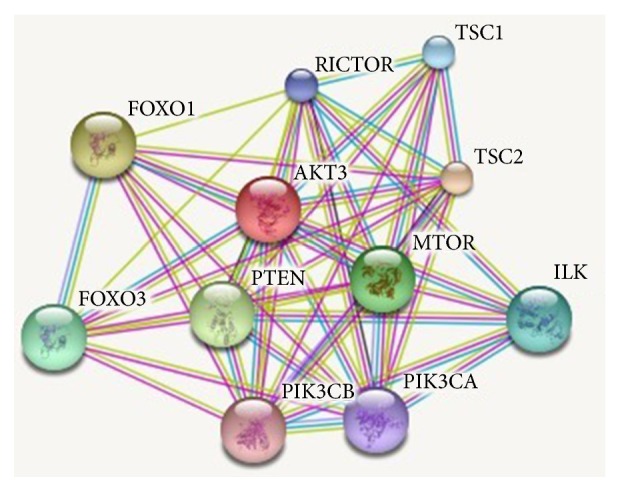
Protein-protein interaction of cow and buffalo* AKT3* gene.

Protein-protein interactions (PPIs) through highly established physical contact and interaction are important for prediction and modulation of the function, activity, and drug ability of the target protein. In PPI of the cow and buffalo, we found different types of indication and interaction. Some of the proteins are known, with predicted 3D structure. Some of the node/protein query indications show the first shell and second shell of interactions. While some of them show the interactions from curated databases and are experimentally determined, the rest of them show the predicted interactions of gene neighborhood, gene fusions, and gene cooccurrences. Some others show text mining, coexpression, and protein homology. The results of PPI were shown in Figure 3 and the data in Supplementary Table 1. There are so many domains found in the cow and buffalo protein sequences with different interval and E values, and some of them are hit by profiles or by patterns (Supplementary Table 2). They have two types of functions. Protein phosphorylation is biological process and the molecular function, which are characterized in three categories (protein kinase activity, protein serine/threonine kinase activity, and ATP binding). Some software tools show that their predicted features are ATP, protein kinase, and protein acceptor.

### 3.6. Phylogenetic, Alignment, Structural, and Motif Analysis of* AKT3* Gene

**Figure 4:**
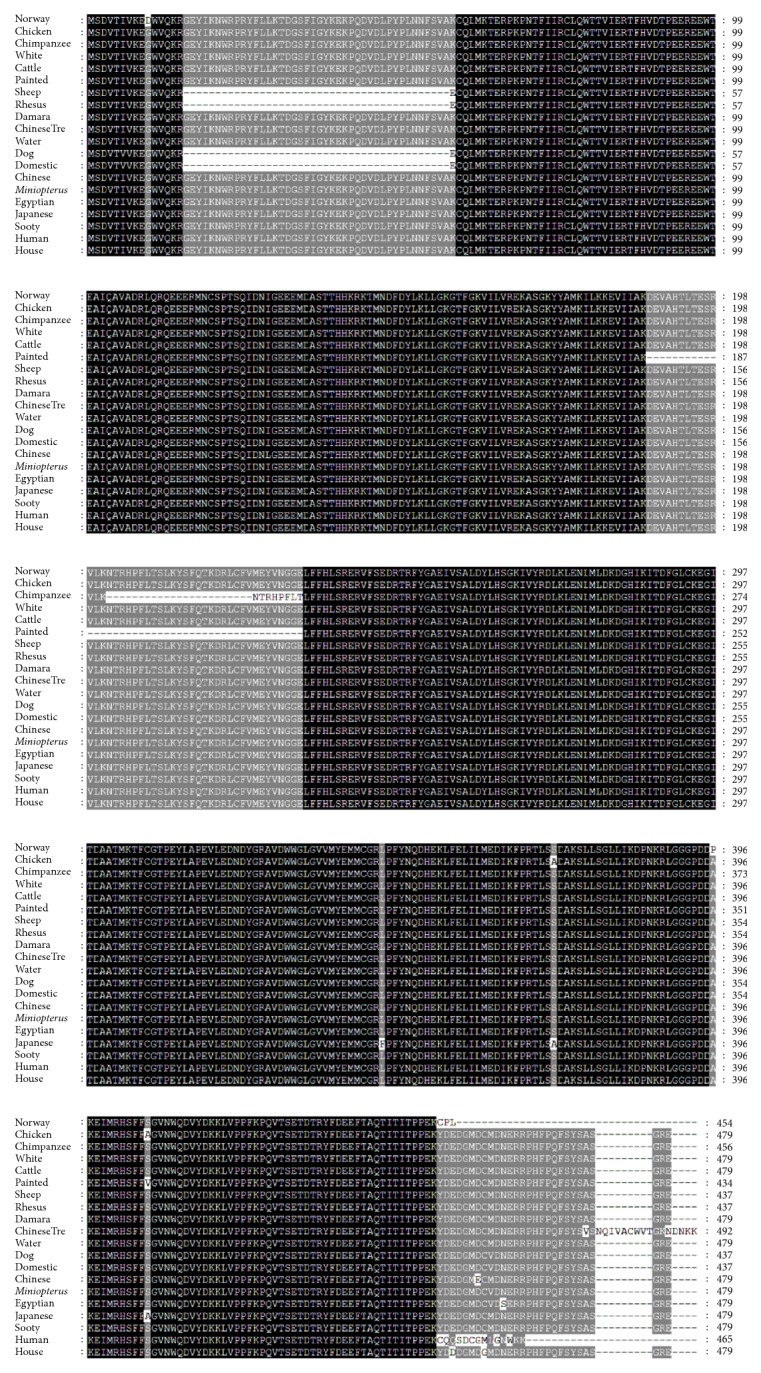
A multiple sequence alignment (MSA) of* AKT3* gene in 20 different species. The different color shows the conserved regions in 20 different species. The deep black strip lines which consist of mixtures of colors of green, red, yellow, and so on indicate high intensity with 100% conserved region similarity, the light black color indicates more than 75% intensity of conserved region in these species, and the white color shows 50% of conserved region similarity.

**Figure 5:**
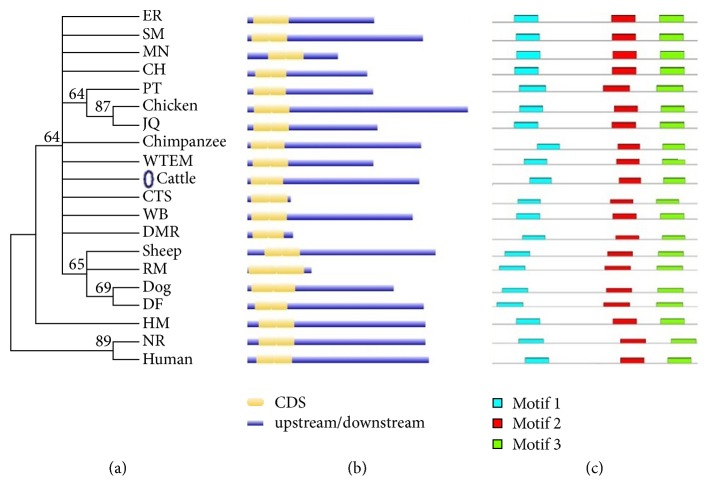
Phylogenetic relationship, gene structure, and motifs composition of* AKT3* gene in 20 different species. (a) Phylogenetic trees by the neighbor-joining method constructed by the multiple alignments of 20 full length proteins of* AKT3* gene with the MEGA 6.0 software. (b) Gene structure for 20 different species using GSDS. (c) Schematic conserved motifs representation of the* AKT3* gene by MEME software tool. Each motif is represented by a colored box. The nonconserved sequences are represented by black line.

Multiple sequence alignment (MSA) is a technique of bioinformatics to find out the region of important similarity in different sequences, which may indicate the evolutionary, structural, and functional relationship in the biological sequences and homologous regions of different sequences. Phylogenetic analysis and a MSA were performed using quick tree. The evolutionary tree is finally designed with MEGA6 or FigTree. Phylogeny or evolutionary tree or phylogenetic tree is a branching diagram based on the information of difference and similarity, genes from common ancestor, and evolutionary relationship among various species. In this analysis we used the proteins sequences from 20 different species. The similarity of the different species was described in Supplementary Figure 1. This shows different similarity score, in the way of assessing the evolutionary liaison of* AKT3* by means of further genus, neighbor-joining methods used for constructed phylogenetic trees based on amino acid sequencing of* AKT3*. The phylogenetic trees showed the different group of species with close genetic relationships. The* AKT3* was much conserved in different species and clustered in different groups. Based on the phylogenetic tree, the multiple alignments are also performed for these 20 different species and the results are shown in Figure 4. We have analyzed in depth the structure, distribution, and conserved motifs of the* AKT3 *in the 20 different species according to the phylogenetic relationship and the results are given in Figure 5(b). Pattern of widespread amino acids or nucleotide sequence with biological importance is called motifs. To examine the diverse structure of AKT3 protein, we used the MEME program supported by InterPro subsequent annotation. The conserved motifs were identified in all tested species. The conserved motifs are displayed and each is represented by colored box and the nonconserved sequence is presented by black lines (Figure 5(c)). The CPG island was also checked for both cow and buffalo for* AKT3* gene and the results are shown (Supplementary Figure 2).

## 4. Conclusion

Our findings show that there are substantial differences of* AKT3* sequences and positions between the buffalo and cow. This may, at least in part, explain the variation, potential resistance, and higher immunity against hostile environments and diseases in buffalo compared with that in cows. Their upstream of the ATG, “3′UTR,” and CDS are substantially different which may be related to the differences in initiation of transcription and gene expression in different tissues. The important consensus sequences motifs differences have been found between the buffalo and cow at different positions. The gene structures are also substantially different between buffalo and cow. Our study provided information for genetics selection for disease and environment resistance in these species although other factors should be considered. Further studies, such as association of these differences and variation with disease and environment resistance between buffalo and cow for* AKT3 *gene, are required to confirm our findings.

## Figures and Tables

**Table 1:** SNPs list of cow AKT3 reported on NCBI and Ensembl databases.

S. no.	SNP/db alleles	ID/variation	Chromosome position	A. acids position	mRNA position	A.A/protein residues	Codon' position	Function
1	G/A	rs444696404	34132704	6	57	Val/lle	1	Missense
2	T/A	rs453731244	34132715	9	68	Asp/Glu	3	Missense
3	T/A	rs466273534	34260902	21	103	Lle/Asn	2	Missense
4	G/T	rs456763634	34292681	82	287	Val/Val	3	Synonymous
5	T/G	rs457591403	34298319	96	327	Ter/Glu	1	Nonsense
6	G/C	rs471195960	34298329	99	337	Arg/Thr	1	Missense
7	G/T	rs439806731	34298347	105	355	Gly/Val	2	Missense
8	G/A	rs459854764	34298351	106	359	Ala/Ala	3	Synonymous
9	T/G	rs480000100	34298378	115	386	Asp/Glu	3	Missense
10	C/A	rs442086028	34298382	117	390	Leu/Met	1	Missense
11	C/A	rs462167315	34298402	123	410	Ser /Ser	3	Synonymous
12	G/A	rs482260846	34298406	125	414	Val/lle	1	Missense
13	A/G	rs449902570	34298418	129	426	Arg/Gly	1	Missense
14	C/A	rs469862137	34298428	132	436	Ala/Glu	2	Missense
15	T/A	rs477028903	34298430	133	438	Leu/Met	1	Missense
16	G/C	rs445770330	34298447	138	455	Thr/Thr	3	Synonymous
17	T/G	rs433341688	34321002	191	614	Ala/Ala	3	Synonymous
18	T/G	rs472740488	34345952	240	761	As/Glu	3	Missense
19	-/C	rs524264518	34353289	315	984	Gln/Pro	1	Frameshift
20	G/T	rs478184191	34363705	317	991	Gly/Val	2	Missense
21	G/A	rs446912876	34363709	318	995	Leu/Leu	3	Synonymous
22	T/C	rs210226650	34363778	341	1064	Cys/Cys	3	Synonymous
23	G/A	rs435659650	34363814	353	1100	Lys/Lys	3	Synonymous
24	C/A	rs449234490	34363880	375	1166	Ser/Ser	3	Synonymous
25	A/T	rs469391365	34363881	376	1167	Met/Leu	1	Missense
26	C/T	rs437087303	34363897	381	1183	Ser/Leu	2	Missense
27	G/T	rs457180310	34363913	386	1199	Lys/Asn	3	Missense
28	T/A	rs464373518	34363915	387	1201	Lle/Lys	2	Missense
29	C/A	rs460962702	34404272	452	1396	Ser/Tyr	2	Missense
30	G/T	rs480978676	34404273	452	1397	Ter/Tyr	3	Missense
31	G/A	rs449685572	34404275	453	1399	Gly/Asp	2	Missense
32	T/G	rs464085150	34404283	456	1407	Cys/Gly	1	Missense
33	G/T	rs477739707	34404285	456	1409	Gly/Gly	3	Synonymous
34	C/T/G	rs446248358	34404288	457	1412	lle/lle	3	Missense
35	C/A	rs466346677	34404302	462	1426	Thr/Asn	2	Missense
36	T/G	rs479967584	34404308	464	1432	Met/Arg	2	Missense
37	T/G	rs448466150	34404309	464	1433	Ser/Arg	3	Missense
38	A/C	rs468424365	34404310	465	1434	Arg/Arg	1	Synonymous
39	C/A	rs134889910	34404317	467	1441	Pro/His	2	Missense
40	A/C	rs457167608	34404318	467	1442	Gln/His	3	Missense
41	G/T	rs470108761	34404319	468	1443	Val/Phe	1	Missense
42	T/C	rs432214133	34404321	468	1445	Phe/Phe	3	Synonymous
43	A/G	rs452216512	34404327	470	1451	Gln/Gln	3	Synonymous
44	A/C	rs472398822	34404336	473	1460	Ter/Tyr	3	Nonsense
45	C/T	rs717060180	34404339	474	1463	Ser/Ser	3	Synonymous
46	A/G	rs440955753	34404340	475	1464	Thr/Ala	1	Missense

**Table 2:** List of substantial differences between cow and buffalo in exon regions, traced by mRNA sequences of cow and buffalo for confirmation.

S. no.	Exon	Variation/mutation	mRNA position	AA position	AA changed	Changes
1	4	A/G	365	122	D/G	Aspartic acid/glycine
2	6	C/T	617	206	T/I	Threonine/isoleucine
3	7	A/G	704	235	Y/C	Tyrosine/cysteine
4	9	A/G	971	324	Q/R	Glutamine/arginine
5	12	A/G	1322	Yes441	H/R	Histidine/arginine
6	13	G/C	1593	Yes531	P/P	Proline/proline
7		T/C	1617	Yes539	P/P	Proline/proline
8		G/A	1623	Yes541	P/P	Proline/proline
9		T/C	1643	Yes548	I/T	Isoleucine/threonine

**Table 3:** List of important consensus sequence motifs and variations observed for AKT3 of cow and buffalo.

Motifs	Consensus sequence	Regions	Cow sequence	Regions	Buffalo sequence
TATA sequence	TATA	−1267 to −1271, −1328 to −1332, −1365 to −1369, −1863 to −1867, −1927 to −1931,	TATA	−647 to 651, −656 to −660, −1144 to −1148, −1263 to −1267, −1324 to −1328, −1359 to −1363, −1367 to −1371, −1858 to −1862, −1906 to −1710, −1922 to −1926,	TATA
	TATAA	−649 to −654, −1372 to −1377, −1536 to −1541, −2225 to −2230, −2289 to −2294, −2657 to −2662,	TATAA	−646 to −651, −1366 to −1371,	TATAA
	TATAAA	−1145 to −1151, −275 to −280,	TATAAA	N/A	TATAAA
	TATAA changed to	−632 to −6637, −658 to −663, −677 to −682, −733 to −738, −805 to −810, −834 to −839, −860 to −864, −1053 to −1058, −1076 to −1081, −1218 to −1223, −1277 to −1282, −1336 to −1341, −1507 to −1512, −1813 to −1818, −1904 to −1909,	TGTAA, TGTAA, TGTAA, TTTAA, TTTAA, TGTAA, TGTAA, TTTAA, TGTAA, TTTAA, TGTAA, TTTAA, TCTAA, TCTAA, TTTAA,	−275 to −280, −629 to −634, −674 to −679, −730 to 735, −802 to 807, −831 to −836, −858 to −863, −1050 to −1055, −1214 to 1219, −1273 to −1278, −1332 to 1337, 1501 to − 1506, −1807 to −1812, 1899 to −1904,	TGTAA, TGTAA, TTTA A, TTTAA, TGTAA, TG TAA, TTTAA, TGTAA, TGTAA, TTTAA, TCTAA, TCTAA, TTTAA, TTTAA,

CRE	TGACGTCA	−219 to −227, −1661 to −1669,	TGAGGTCA, TGACATGA	−204 to −212, −219 to −227, −1677 to −1685,	TGAAGACA, TGAGGTCA, TGAGGACA,

E-box	CANNTTG	−24 to −31, −245 to −252, −901 to −908,	CAAGTTG, CAGTTTG, CAGATTG,	−24 to −31, −245 to −252, −898 to −905,	CAAGTTG, CAGTTTG CAGATTG,

EC	GTGG	−95 to −99, −368 to −372, −918 to −922, −950 to −954, −988 to −992, −1288 to −1292, −1652 to −1656,	GTGG	−95 to −99, −367 to −371, −915 to −919, −947 to −951, −955 to −989, −1284 to −1288, −1646 to −1650,	GTGG

CAAT	CAAT	−130 to −134, −398 to −402, −525 to −529, −541 to −545, −690 to −694, −826 to −830, −1271 to −1275, −1379 to −1383, −1384 to −1388,	CAAT	−130 to −134, −397 to −401, −524 to −528, −540 to −544, −687 to −691, −823 to −827, −1267 to −1271, −1373 to −1377, −1378 to −1382,	CAAT

NF-KB	GGGRNNYY CC	−3 to −13, −14 to −24, −82 to −92, −847 to −857, −1422 to −1432,	GGGAGCCATC, GGGGCTCAGC, GGGCAGCAGC, GGGAATCAGC, GGGTTTTTCC,	−3 to −13, −82 to −92, −844 to −854,	GGGAGCCATC, GGGCAGCAGC,

IRF	GAAANNGA AAG	−1036 to −1048, −1539 to −1551	GAAACTGATTTT, GAAAAAGAACTA	−1033 to −1045, −1533 to −1545,	GAAACTGATTTT, GAAAAAGAACTG

SP-1 site	GGGCGG	−10 to −16, −19 to −25, −85 to −92, −916 to −922, −1652 to −1659,	GCGGGG, GGGGGC, GGGCAG, GTGGGC, GGGGTGG,	−6 to −16, −19 to −25, −85 to −92, −913 to −917, −1132 to −1136, −1646 to −1653	GAGGGGAGCC, GGGGGC, GGGCAGC, GGGC, CGGG,GGGGT GG,

TSS		−95, GGGAAG, −1795, ATTTTT	G, T	−165, TTGCAA −1765, TCTGTC	G, T

CPG island	N/A			N/A	

Predicted promoters		−225 to −275, −826 to −876,	Identity 100, but showing variation at one position as G/T	−225 to −275, −823 to −873,	

**Table 4:** List of variations found in the 2000 bp of the cow and buffalo in the upstream region from starting ATG.

S. no.	Variation	Changes
1	-/A	->A-1993
2	G/A	G>A-1989
3	T/C	T>C-1891
4	A/T	A>T-1830
5	-/C	->C-1821
6	C/T	C>T-1555
7	A/G	A>G-1537
8	T/G	T>G-1536
9	T/G	T>G-1532
10	T/C	T>C-1422
11	A/G	A>G-1404
12	T/-	T>-1366
13	A/-	A>-1365
14	T/C	T>C-1252
15	A/G	A>G-1198
16	T/-	T>-1176
17	C/A	C>A-1087
18	A/G	A>G-1086
19	A/T	A>T-1075
20	C/A	C>A-891
21	C/T	C>T-881
22	G/T	G>T-871
23	C/T	C>T-812
24	C/T	C>T-782
25	A/G	A>G-755
26	C/T	C>T-744
27	T/C	T>C-723
28	C/A	C>A-704
29	G/A	G>A-659
30	A/T	A>T-656
31	C/A	C>A-636
32	C/-	C>-568
33	A/-	A>-567
34	G/A	G>A-507
35	C/T	C>T-452
36	T/C	T>C-450
37	C/A	C>A-404
38	A/-	A>-358
39	A/G	A>G-308
40	C/A	C>A-12

**Table 5:** List of miRNA predicted in the “3′UTR” region of cow and buffalo.

S. no.	Accession	ID	Query start	Query end	Subject start	Subject end	Strand	Score	*E* value	Alignment	Species
1	Mimat0024577	bta-miR-574	163	184	2	23	−	65	5.4	Align	Cow
2	Mimat0012060	bta-miR-2470	103	118	6	21	−	62	9.7	Align	

1	Mimat0011984	bta-miR-1814c	932	949	3	20	+	72	5.1	Align	Buffalo
2	Mimat0024577	bta-miR-574	162	184	2	24	−	70	7.4	Align	
3	Mimat0012021	bta-miR-2444	538	554	4	20	−	76	2.3	Align	
4	Mimat0011874	bta-miR-1814a	533	548	5	20	−	71	6.1	Align	
5	Mimat0011946	bta-miR-2393	533	548	5	20	−	71	6.1	Align	
6	Mimat0012011	bta-miR-2437	533	553	1	21	−	69	9.0	Align	
7	Mimat0011934	bta-miR-2325c	380	399	1	20	+	73	4.2	Align	
8	Mimat0011943	bta-miR-2390	381	400	1	20	+	73	4.2	Align	
9	Mimat0012011	bta-miR-2437	377	397	1	21	+	69	9.0	Align	

## Supplementary Materials

Supplementary Materials The supplementary file contains two supplementary figures and two supplementary tables. Supplementary Figure 1: phylogenetic relationship of 20 different species for the* AKT3* gene. Supplementary Figure 2: CPG island for the* AKT3* gene in buffalo and cow using two different software tools. Supplementary Table 1: predicted protein-protein interaction of cow and buffalo* AKT3*. Supplementary Table 2: list of predicted domains in cow and buffalo for* AKT3* gene.
