# Supplementary material for: Comparative Analysis of V-Akt Murine Thymoma Viral Oncogene Homolog 3 (AKT3) Gene between Cow and Buffalo Reveals Substantial Differences for Mastitis
Source: Biomed Res Int. 2018 May 15;2018:1463732. doi: 10.1155/2018/1463732 (PMC5976927; doi:10.1155/2018/1463732)
Supplement: Supplementary Materials — The supplementary file contains two supplementary figures and two supplementary tables. Supplementary Figure 1: phylogenetic relationship of 20 different species for the AKT3 gene. Supplementary Figure 2: CPG island for the AKT3 gene in buffalo and cow using two different software tools. Supplementary Table 1: predicted protein-protein interaction of cow and buffalo AKT3. Supplementary Table 2: list of predicted domains in cow and buffalo for AKT3 gene. [file 1463732.f1.docx]

**Figure-1**

**
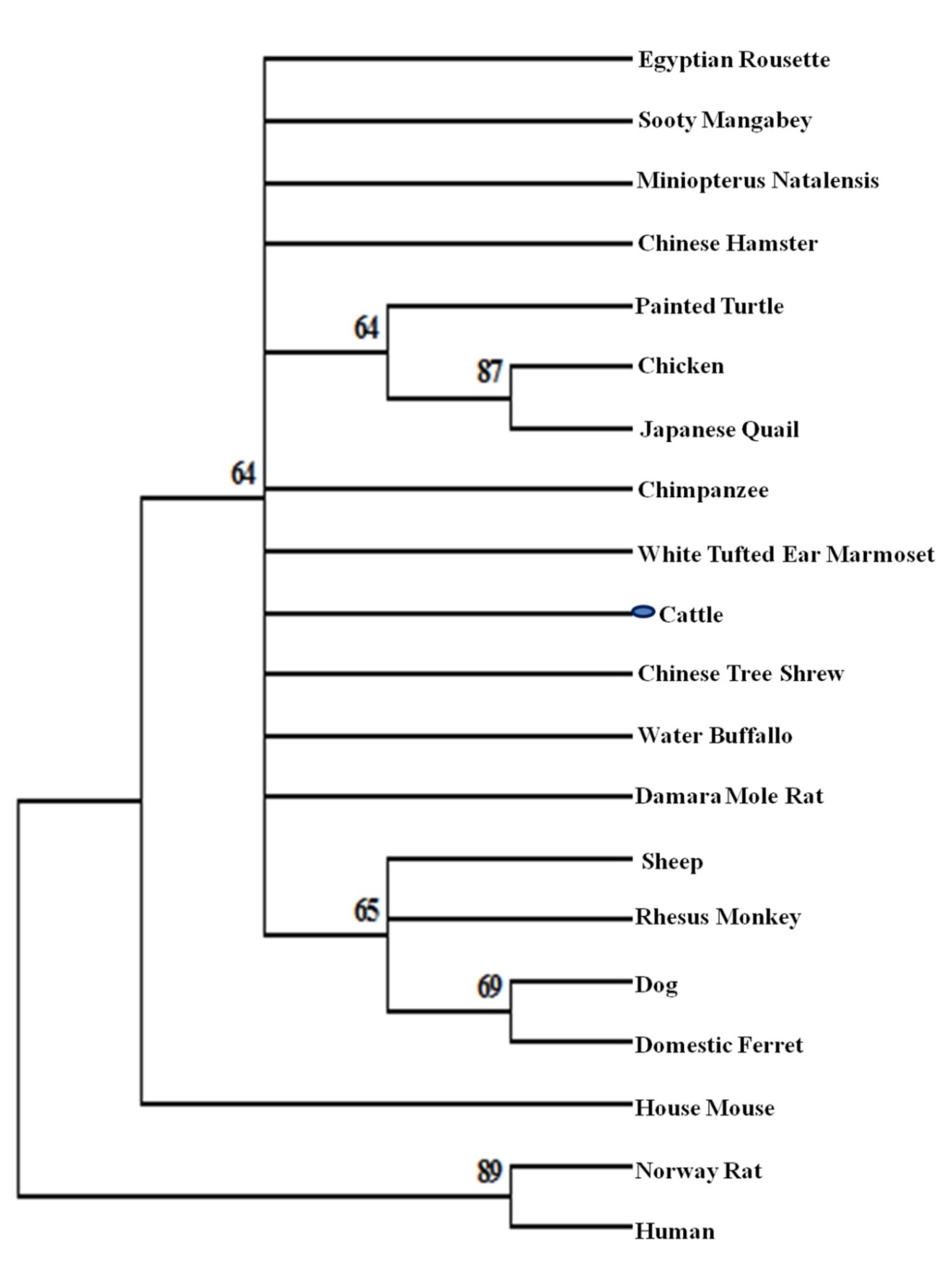
**

**Figure-2**

**
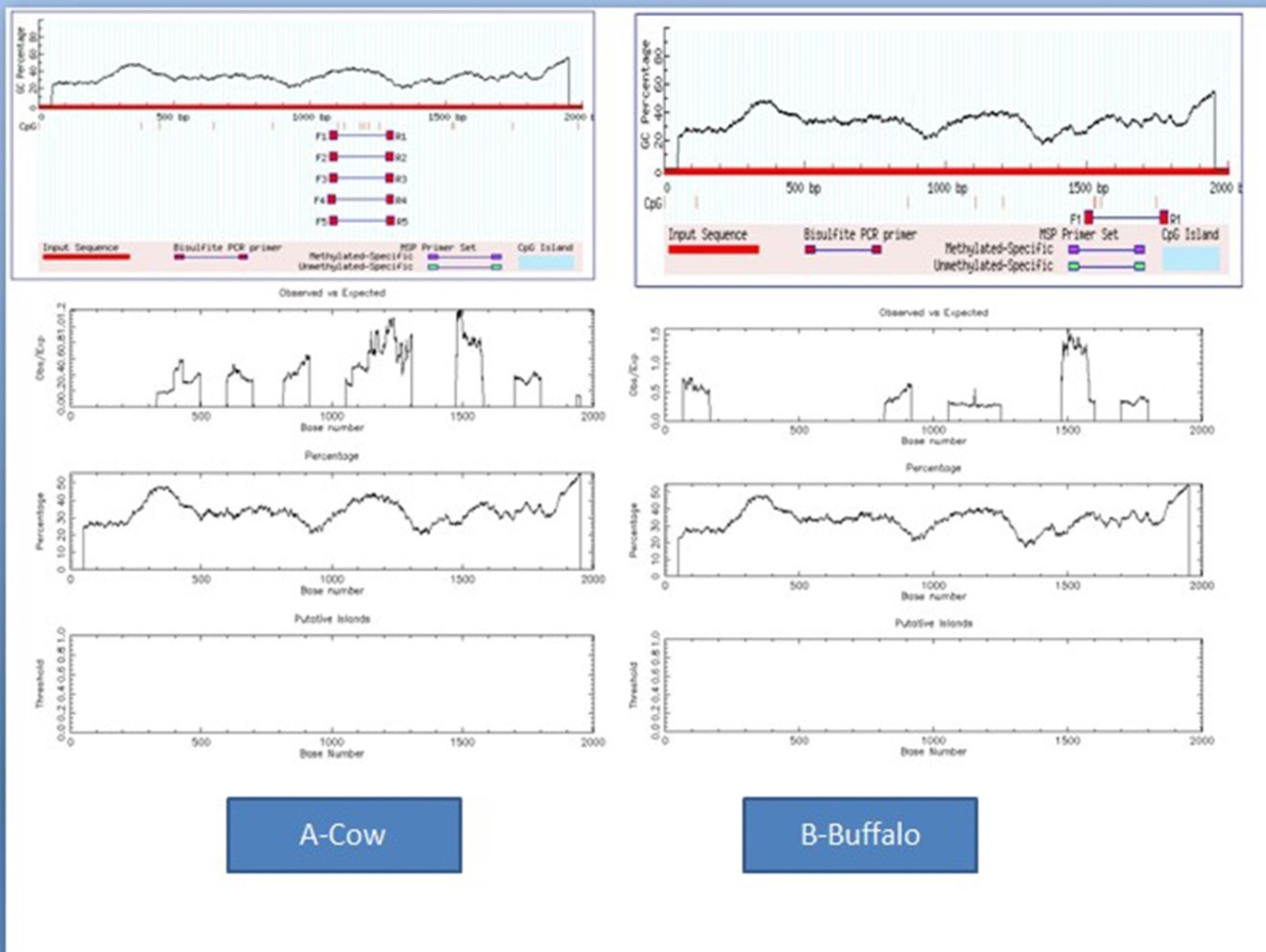
**

**Table No.1-** Predicted Protein-protein interaction of cow and Buffalo AKT3 gene

| S.No | Predicted Functional Partners | Type | AA | Score |
| --- | --- | --- | --- | --- |
| 1 | TSC2 | Uncharacterized Protein | 1814aa | 0.998 |
| 2 | FOXO1 | Uncharacterized Protein |  | 0.997 |
| 3 | PTEN | Uncharacterized Protein | 369aa | 0.997 |
| 4 | MTOR | Uncharacterized Protein | 2551aa | 0.996 |
| 5 | FOXO3 | Forkhead box protein 3 | 672aa | 0.994 |
| 6 | ILK | Integrin linked protein Kinase |  | 0.991 |
| 7 | TSC1 | Uncharacterized Protein | 1160aa | 0.990 |
| 8 | RICTOR | Rapamycine-insensitive companion of mTOR | 1708aa | 0.987 |
| 9 | PIK3CA | Phosphatidylinositol 4,5-biphosphate 3-kinase catalytic subunit alpha isoform |  | 0.987 |
| 10 | PIK3CB | Phosphatidylinositol 4,5-biphosphate 3-kinase catalytic subunit beta isoform | 1071aa | 0.986 |

**Table No.2-** List of predicted domains in Cow and Buffalo for *AKT3* gene

| S.NO | Domains | Accession | Interval | Sources | E.Values | Species |
| --- | --- | --- | --- | --- | --- | --- |
| 1 | STKc_PKB_gamma | Sd05593 | 132-479 | NCBI data bases | 0e+00 | Cow |
| 2 | PH_PKB | Cd01241 | 4-110 |  | 8.12e-76 |  |
| 3 | PH domain |  | 6-107 | EMBL-EBI web | 1.3e-14 |  |
| 4 | Protein Kinase Domain |  | 148-405 |  | 2.1e-77 |  |
| 5 | Protein Kinase C Terminal Domain |  | 426-473 |  | 4e-09 |  |
| 6 | PH domain | (hit by profiles) | 5-107 | ScanProsite result viewer web |  |  |
| 7 | Protein Kinase domain |  | 148-405 |  |  |  |
| 8 | AGC_Kinase_CTER |  | 406-479 |  |  |  |
| 9 | Protein_Kinase_ATP | (hit by patterns) | 154-187 |  |  |  |
| 10 | Protein_Kinase_ST |  | 267-279 |  |  |  |
|  |  |  |  |  |  |  |
| 1 | STKc_PKB_gamma | Sd05593 | 90-437 | NCBI data bases | 0e+00 | Buffalo |
| 2 | PH- like super family | cl 17171 | 4-68 |  | 3.11e-33 |  |
| 3 | PH domain |  | 6-107 | EMBL-EBI web | 1.3e-14 |  |
| 4 | Protein Kinase Domain |  | 148-405 |  | 2.1e-77 |  |
| 5 | Protein Kinase C Terminal Domain |  | 426-473 |  | 4e-09 |  |
| 6 | PH_domain | (hit by profiles) | 1-65 | ScanProsite result viewer web |  |  |
| 7 | Protein_Kinase_dom |  | 106-363 |  |  |  |
| 8 | AGC_Kinase_CTER |  | 364-437 |  |  |  |
| 9 | Protein_Kinase_ATP | (hit by patterns) | 112-145 |  |  |  |
| 10 | Protein_Kinase_ST |  | 225-237 |  |  |  |
|  |  |  |  |  |  |  |
